# Spastic wrist flexion in cerebral palsy. Pronator teres versus flexor carpi ulnaris transfer

**DOI:** 10.1590/1413-785220152303145550

**Published:** 2015

**Authors:** Edgard de Novaes França Bisneto, Nivea Rizzi, Eliana Ogassawara Setani, Livia Casagrande, Joseane Fonseca, Glaucia Fortes

**Affiliations:** 1 Department of Orthopedics, Traumatology, Hand Surgery and Microsurgery, Faculdade de Medicina da Universidade de São Paulo, São Paulo, SP, Brazil; 2 Associação de Assistência à Criança Deficiente (AACD), São Paulo, SP, Brazil

**Keywords:** Wrist, Cerebral palsy, Tendon transfer

## Abstract

**OBJECTIVE:**

Analize data on patients submitted to transfer of the pronator teres (PT) or the flexor carpi ulnaris (FCB) to the extensor carpi radialis longus/brevis (ECRL/B) in order to correct flexed wrist deformity in patients with cerebral palsy.

**METHOD:**

Patients were divided into two groups: PT group and FCU group to ECRL/B. The results were evaluated by goniometry and by the functional hand test (FHT).

**RESULTS:**

Goniometry showed a statistically significant difference in favor of FCU transfer. There was no statistically significant difference regarding FHT.

**CONCLUSION:**

Both transfers PT and FCU to ECRB are good options to correct wrist flexion deformity in cerebral palsy. *Level of Evidence III, Non-randomized Controlled Cohort/Follow-Up Study.*

## INTRODUCTION

Cerebral palsy (CP) is a neurological, non-progressive disorder that affects the central nervous system. It is caused by an irreversible, static perinatal brain injury.^1^^,^ 2 Functional activities of the upper limbs are limited in most individuals diagnosed with CP.^3^ These limitations are identified at around one year of age, when the child usually develops the normal pinch grip; in CP, other more primitive pinch patterns may appear.^2^ Surgical interventions are applied in fewer than 20% of pediatric patients whose upper limbs are affected by CP,^3^ but when surgery is chosen, for aesthetic purpose or to facilitate the use of a removable splint, this figure may reach 40% of patients, in our experience.

Despite the fact that all patients with CP have different degrees of spasticity and athetosis,^4^ the most common pattern of upper limb deformities are: Shoulder: internal rotation and adduction; Elbow: flexion; Forearm: pronation; Wrist: flexion and ulnar deviation; Fingers: flexion and Swan-neck deformity; Thumb: adduction and flexion.^1^

The severe flexed position of the wrist decreases the mechanical advantage of the digital flexor tendons, weakening grip strength and precluding normal fingers visual feedback.^2^ This flexed position is the result of spastic flexors and weak extensors, and subsequent capsular contracture.^1^^,^ 3 When non-surgical treatment fails, there are surgical options available to improve wrist extension. A multi-level surgery in one single procedure is preferable to many small procedures,^5^ however this manuscript will focus on correction of wrist flexion deformity.

There are two different surgery goals: soft tissue re-balancing and bone stabilization. Joint stabilization is performed by arthrodesis^2^^,^ 3 ^,^ 6 in non-functional wrists and for cosmesis/hygiene purposes.^2^

Soft tissue surgeries attempt to improve extension and weaken flexor spasticity, in order to provide hand grasp and release.^2^^,^ 3 Many procedures for improving extensor strength have been described^1^^,^ 2 ^,^ 7 ^,^ 8 Transfer of the flexor carpi ulnaris (FCU) to the extensor carpi radialis (ECRL/B) (Green procedure); transfer of the pronator teres (PT) to extensor carpi radialis (ECRL/B); transfer of the extensor carpi ulnaris (ECU) to the extensor carpi radialis (ECRL/B); brachioradialis (BR) to the extensor carpi radialis (ECRL/B). 

In this retrospective paper, the authors reviewed data from patients submitted to pronator teres (PT) or flexor carpi ulnaris (FCU) to extensor carpi radialis (ECRL/B) in order to correct flexed wrist deformity.

## MATERIALS AND METHODS

This work was approved by the Ethics Committee of the Department of Orthopedics and Traumatology, *Faculdade de Medicina da Universidade de São Paulo* under the number CAAE 32947014.6.0000.0085.

From 2008 and 2011, 37 patients diagnosed with cerebral palsy and flexed wrist deformities submitted to surgical correction were included for functional evaluation. Of these, 22 patients were treated surgically with tendon transfer PT to ECRL/B (PT group) and 15 patients were treated with an FCU to ECRL/B tendon transfer (FCU group). 

As a retrospective cohort, there were no criteria for the choice of surgery, and the decision was made by each surgeon, based on his experience.

The inclusion criteria were patients with cerebral palsy GMFCS 1-4, who had undergone FCU or PT transfer to correct flexed wrist deformity.

All the patients were unable to perform any active wrist extension but allowed passive correction to reach neutral position.

Contraindications were: fixed flexed deformities, absence of finger extension and GMFCS (Gross Motor Function Classification System) grade 5 tetraplegic patients. 

All patients were also treated for deformities of the shoulder, elbow and fingers in the same surgical intervention. These concurrent procedures were similar between groups, and did not interfere with the results. (Table 1)

**Table 1.** Usual concurrent procedures

Shoulder	Tenotomy of pectoralis major tendon
Elbow	Biceps to Triceps transfer
	Brachialis lengthening
Wrist	Flexor carpi radialis tendon z- lengthening
	Flexor digitorum superficilais tendon z-lengthening
	Palmaris longus to extensor pollicis brevis transfer
Hand	Tenotomy of the adductor pollicis tendon insertion

There were 16 men and 6 women in the PT group, and 10 men and 5 women in the FCU group. The groups were similar in age: 17.1 (range 10-27) years old in the PT group and 15.6 (range 5-30) years old in the FCU group. The FCU group had more women and the PT group had more men.

Most patients were hemiplegic, Gross Motor Function Classification System (GMFCS) grades 1 to 3 (Table 2) five patients were grade 4 (one patient in FCU group and four patients in PT group). All patients had some kind of upper limb function according to the Manual Ability Classification System^9^ (MACS) grades 1 to 4. (Table 3)

**Table 2.** Gross Motor Function Classification System (GMFCS).

Level I	Walks without limitations
Level II	Walks with limitations
Level III	Walks using a hand-held mobility device
Level IV	Self-mobility with limitations; may use powered mobility
Level V	Transported in a manual wheelchair

**Table 3.** Manual Ability Classification System (MACS).

Level I	Handles objects easily and successfully
Level II	Handles most objects but with somewhat reduced quality or speed of achievement
Level III	Handles objects with difficulty; needs help to prepare or modify activities
Level IV	Handles a limited selection of easily managed objects in adapted situations
Level V	Does not handle objects and has severely limited ability to perform even simple actions

### Surgical Technique

The standard surgical procedure in all patients included lengthening of the flexor carpi radialis, flexor digitorum superficialis, flexor pollicis longus and palmaris longus tenotomy or transfer to extensor pollicis longus/brevis. What differed between the groups was the technique used to correct the flexed deformity. The surgeon chose the appropriate transfer at the time of the surgery, based on his experience. There was no randomization.

Transfer of the Promator Teres (PT): An oblique incision over the transition of the proximal and midportion of the forearm was made. The PT insertion is detached from the radius and attached to the ECRB. The tension in the tendon transfer must balance the wrist after lengthening the flexor digitorum superficialis and flexor carpi radialis and flexor ulnaris tendons. We believe 10° of flexion, with the wrist in the neutral position, is acceptable.

Transfer of the Flexor Carpi Ulnaris (FCU): Through a longitudinal incision of the volar wrist, the FCU is harvested from its insertion in the pisiform. We prefer to make a tunnel around the ulna to improve the supination moment and, through a dorsal incision, reattach the FCU to the ECRB.

Goniometry and the functional hand test (FHT) were performed at six months after surgery in all patients. This represents the point at which our institutional rehabilitation protocol ended.

The FHT is a straightforward test based on the studies by Deaver,^10^ Fusco,^11^ and Carazzato,^12^ which shows good results in a percentage of normal matched-aged hand functions, however this test is not validated in English literature. FHT has been applied in our institution over the last 18 years.

The position of the wrist was measured, with the patient making a fist using a goniometer placed along the dorsal forearm and third metacarpal. The zero position was taken as 90° of wrist flexion. Thus, the neutral wrist position is recorded as 90°; therefore, 90° of wrist extension is recorded as 180°.

### Statistical analysis

The results were arranged in three different subgroups for each procedure. In the PT-ECRB group the results are arranged in the following subgroups: patients younger than 12 years old; patients older than 12 years old and all patients. The same was done in the FCU-ECRB group. 

The data were analyzed by the ANOVA (analysis of variance) parametric test. Comparison between results in each group was done by the Student *t*-test. For this analysis, we considered α≤0.05.

## RESULTS

Table 3 shows the gender distribution in the two groups. According to goniometry tests, all patients had 90° flexed wrists with no active extension prior to the surgery. 

After the surgery, there was a statistically significant difference between groups (p=0,041) in favor of FCU transfer regarding maximal active extension. The FCU group showed wrists in mild extension, 6.7-7.5 degrees on average, and the PT group patients showed mild flexion, 18.7-16.1 degrees of flexion on average.

We also noticed that the younger the patients, the more similar the results between the techniques. Considering only patients younger than 12 years old (pre-adolescents) there was no difference (p=0.3) in goniometry between the transfer techniques used. When we included patients aged between 12 and 15 years old, the values were still similar, but were closer to values of the entire patient group, with more individuals than the group under 12 years old. In the entire patient group, there were differences between the results, with better results for the FCU transfer. (Table 4)

**Table 4.** Comparison of goniometry measurements in degrees considering ages groups.

Goniometry	Up to 12 years old		12-15 years old		All patients	
	FCU	PT	FCU	PT	FCU	PT
Number of individuals in each group	3	3	6	16	15	22
Average of active extension	93.3	90.0	96.67	74.06	96.7	73.9
Median of active extension	90	90	90	90	90	90
Standard deviation	5.8	0	33.27	38.61	22.6	37.1
Minimum active extension	90	90	60	0	60	0
Maximum active extension	100	90	150	130	150	130
*p*-value	0.374		0.221		0.041	

There was no statistically significant difference between the groups regarding the FHT before and after surgery (49.8% in the FCU group and 45.7% in the PT group), but both groups had significant improvements in hand function. (Table 5)

**Table 5.** Functional Hand Test results before and after surgery*.

FHT	FCU		PR	
	Before	After	Before	After
Average value	34.5	51.2	37.3	47.5
Median	31	50	40	52
Standard deviation	15.0	14.4	19.9	17.7
Minimum value	4	23	5	12
Maximum value	53	85	64	78
Number of patients	13	13	18	18
*p-*value	<0.001		<0.001	

* Two patients in FCU group and four patients in PT group did not perform the FHT after surgery.

Considering the age groups: Under 12 years old, 12-15 years old, and all patients, the results were similar, with no statistically significant differences between them regarding pre *versus* post-operative functional results measured by FHT. There were no complications in our cohort study. 

## DISCUSSION

FCU to ECRB transfer, also known as Green procedure, is considered the gold standard treatment for flexed wrist by many authors.^1^^,^ 3 ^,^ 6 In the authors' practice, PT transfer is an alternative procedure to correct flexed wrist deformity. In this paper, the authors made a retrospective analysis, comparing the results of PT transfer with those of FCU transfer.

Pronator teres transfer improves wrist extension and shows good results in up to 80% of patients.^13^

PT transfer improves wrist extension and can be used as an alternative method, particularly when the FCU is used as a digit extender.^1^^,^ 2 ^,^ 7 Harvesting the spastic PT can sometimes improve supination as well, but this fact remains controversial.^8^^,^ 14 Restoring supination was not a goal, since under cultural considerations, the neutral position of the forearm is accepted in our population. 

There are some concerns that the flexed wrist could become a rigid dorsiflexed wrist or fixed supination forearm deformity after PT to ECRB transfer. This over correction complication did not occur in our cohort.

An evaluation was carried out six months after surgery by goniometry and by the functional hand test (FHT).^10^^-^ 12 This time period was chosen because it represents the end of our institutional rehabilitation protocol. Goniometry showed 6.7° (on average) of maximum active extension in the FCU group. Active extension did not reach the neutral position in the PT group, with 16.1° of flexion. 

The functional hand test (FHT) improved in both transfers, 44% (range 34.5 to 49.8%) in the FCU group and 22% (range 37.3 to 45.7%) in the PT group (p-value <0.001). There was no statistically significant difference between groups. The FHT values show that the surgery improved hand function. This is an invalidated international test, which is a weakness in our study, but it is an established test at our institution. The patients were not submitted to any other objective functional test.

In the authors' practice, they observed that the younger the patients, the more similar the results between FCU and PT transfers. However, in this cohort, there was no statistically significant difference between age groups, perhaps due the small number of individuals in the group under 12 years old.

Pronator teres transfer is the gold standard treatment for restoring wrist extension in radial nerve palsy, since this muscle is capable of extending the wrist. Alternatively, we can use PT transfer with a tenodesis effect to avoid wrist flexion.

PT transfer may be relatively weaker than FCU transfer in adults with cerebral palsy, or the presence of joint contracture may be part of the physiopathology of the wrist deformity.^15^ In this study, PT was not able to promote active extension of the wrist, even with total passive range of motion. Other reasons for the lack of extension in the PT group could be rupture of the suture in some cases, poor positioning of the wrist, or ECRB adhesion. The authors also consider that only a clinical evaluation of the spastic PT may not be sufficient to consider it a good option for transfer. Preoperative dynamic Electromyography (EMG) is not available at our institution. Perhaps a better analysis of PT condition should have been done, but this also does not explain why PT did not have the tenodesis effect in some adults. 

All patients were submitted to similar concurrent procedures, therefore we do not consider that flexor lengthening had any effect on the outcome of the wrist tendon transfer. There were cases of FCU transfer to the extensor digitorum communis (EDC) in this cohort.

At the time of evaluation, six months after surgery, if active extension was not achieved, the authors did not consider it a recurrence deformity, but rather a treatment failure. Future evaluations will be able to recognize recurrences.

We wish to point out some weaknesses in this paper: the small number of individuals in the group under 12 years old, which severely limited the power of any statistical conclusion, and the fact that the FHT is not a validated international test.

## CONCLUSION

FCU to ECRB tendon transfer is the most reliable option for correcting wrist flexion deformity in cerebral palsy. Pronator teres transfer is a good option in the absence of FCU transfer.
